# The influence of outdoor play spaces in urban parks on children's social anxiety

**DOI:** 10.3389/fpubh.2022.1046399

**Published:** 2022-12-06

**Authors:** Yu Bao, Ming Gao, Dan Luo, Xudan Zhou

**Affiliations:** ^1^Jilin Provincial Key Laboratory of Tree and Grass Genetics and Breeding, College of Forestry and Grassland Science, Jilin Agricultural University, Changchun, China; ^2^Key Laboratory of Cold Region Urban and Rural Human Settlement Environment Science and Technology, Ministry of Industry and Information Technology, School of Architecture, Harbin Institute of Technology, Harbin, China; ^3^Key Laboratory of New Technology for Construction of Cities in Mountain Areas, School of Architecture and Urban Planning, Chongqing University, Chongqing, China

**Keywords:** urban park, play space, children, social anxiety, children's health

## Abstract

Urban green spaces are critical to the healthy development of children's social interactions and activities. However, the relationship between urban green spaces for children's activities and the alleviation of children's social anxiety remains unclear. In this study, we aimed to explore the link between spatial characteristics and social anxiety in children. To explore the coupling relationships among green space, play space, and social anxiety, an assessment of children's play spaces and levels of social anxiety was performed using self-reported data, OpenStreetMap captured the characteristic indicators of urban parks, and the ArcGIS and SPSS softwares were used for the mathematical analysis. The results showed that (1) both the NDVI and 10-min accessibility were significantly negatively related to children's social anxiety; (2) the diversity of service facilities, surfacing materials, and the attractiveness and challenge of the environment were negatively related to children's social anxiety; (3) there were significant differences between activity types and social anxiety. These results provide design references and a theoretical basis for improving the benefits of urban green space on children's health and wellbeing.

## Introduction

Social anxiety disorder (SAD) is the most common type of anxiety disorder that contributes to other mental health disorders (1, 2) such as anxiety and depression (3). An estimated 9–46.7% of young people suffer from anxiety symptoms (4, 5), and the level of social anxiety among young people is rising (6). Social impairment in children predicts higher levels of depression (7). Prejudiced perceptions of social functioning in socially anxious children result from repeated exposure to unpleasant social interactions or a lack of positive interactions (8). Socially anxious children's biased perceptions of social functioning are the result of repeated exposure to unpleasant social interactions or a lack of positive interactions. Studies have found that young people perform poorly in social situations, especially in negative interactions with peers, and this poor outcome, in turn, leads to anxiety, which, in turn, provides fewer opportunities for social skills, leading to poorer social performance (9). If not taken seriously, childhood social anxiety typically persists into adolescence or adulthood and increases the risk of psychological disorders (10, 11). SAD in childhood is associated with a wide range of negative outcomes, including disruption of social relationships, physical symptoms, substance abuse, and other psychiatric problems (12, 13). The main research tool used to assess social anxiety is the Social Anxiety Scale for Children-Revised (SASC-R) (14), which is a widely used self-report instrument of children's subjective feelings of social anxiety that has been used to assess the clinical and symptomatic levels of children's social anxiety (15).

With the advancement of urbanization, the benefits of green space have also been magnified. The impact of urban green spaces on physical and mental health has been extensively demonstrated (16). Research theories supporting this conclusion include the attention restoration theory (ART) (17), stress recovery theory (SRT) (18), and the biophilia theory (19). Among these theories, the SRT considers a non-threatening natural environment to be a restorative environment, which promotes improving people's positive emotional state and offers the best physiological arousal level (20). Some studies have found that a natural environment, no matter what type, can increase psychological wellbeing, including positive emotions, subjective life satisfaction, positive affect, subjective life satisfaction, and meaning in life (21). The ART describes natural settings as having soft fascination, in which nature can hold people's attention, thus, resting executive systems that regulate directed attention, holding back pessimistic thoughts, and reducing or eliminating negative emotions to be replaced by positive emotions (22). For example, studies have shown that bird songs and water sounds in nature have obvious positive regulating effects on children's stress recovery (23). According to the biophilia theory, the most basic human need is to communicate with nature (24). For example, when people hear words such as forest, green space, landscape, and wildflowers, it has a positive effect on improving mental health and life satisfaction (19). Even brief contacts with natural environments such as gardens and green spaces can improve psychological states (25) and are critical to “public mood” (26). Contact with a natural environment has a positive effect on children's mental health (27, 28) and also leads to better cognitive, mental health, and immune function (29). Nature exposure—in particular, the amount of physical contact that an individual has with nature (30) in childhood—is thought to develop habits, preferences, and mental health (31, 32). More nature exposure during childhood is thought to foster better habits, preferences, and mental health (32), for example, providing more nature exposure in daily life leads to a greater understanding of the biosphere and greater empathy for other organisms (33). In addition, the level of nature exposure during childhood is positively correlated with children's perceptions of the natural world as adults, as well as influencing their environmental behavior and awareness of environmental issues (34). Therefore, more outdoor activities in urban parks contribute positively to children's physical and mental development. Outdoor play areas can also develop children's language and comprehension, physical activity, and social skills (35, 36). This confirms the link between activity space design and value (37, 38). At the same time, children's positive or supportive interactions with playmates may be a protective factor that can help socially anxious children to develop more realistic views of their social functioning (38). However, the intrinsic link between urban green space characteristics and children's social anxiety is still not sufficiently understood.

In addition, children prefer to play with friends and partners when engaging in outdoor activities (39). Social distance, as proposed by research scholars, has been defined as the degree to which an individual is willing to interact or spend time with others outside of his or her own group (40). At the same time, social distance has been used as an important tool to assess the degree of closeness between individuals (41). Therefore, the distance between children and each other should be considered. Moreover, studies have found that social distance included close distance, individual distance, social distance, and public distance. When participating in activities with friends, adults' social distances are typically within 1.2–3.5 meters (42). These scales are often more appropriate for the design of specific elements within a space. This can provide a more rational and effective basis for the design of the interior of children's activity spaces.

Landscape features, facilities, amenities, and maintenance in urban parks indicate the quality of a green space (43). Various relationships between health and urban green space characteristics have been proposed, such as area size (44), space type (45), vegetation cover (46), and the normalized difference vegetation index (NDVI) (47) (the most commonly used plant cover index) that reflects plant growth, vegetation type and biomass, and is linearly related to vegetation cover (48). Urban parks provide places for children to play, especially in highly urbanized environments, and are essential outdoor resources for children's wellbeing (49, 50). The focus of urban planning has been to build child-friendly cities that promote children's development. Children's playgrounds should be fully integrated into urban open space systems and should be evenly distributed and easily accessible (51). Therefore, many studies have proposed that accessibility is the criterion to measure whether an urban space is child oriented (52), while, at the same time, evaluating the fairness and suitability of services provided by urban green spaces.

In this study, we aimed to investigate the effect of urban green spaces on children's social anxiety. We analyzed the relationship between children's social anxiety and spatial environments and explored the need for spatial environments to alleviate the level of social anxiety. In this work, we ask the following research questions:

**Research Question 1 (RQ1)** Is there a significant relationship between urban green space characteristics and children's social anxiety?
**Research Question 2 (RQ2)** If the answer to RQ1 is positive, is there a significant relationship between playing fields in green spaces and children's social anxiety?
**Research Question 3 (RQ3)** If the answer to RQ2 is positive, then, do the characteristics of children's activities in the playing field affect the patterns that have been observed?

## Materials and methods

### Study area

The study was conducted in Changchun, Northeast China, which is a developing megacity in China, with a population of more than 8 million. The urbanization level is relatively high (53), and the urban green coverage rate exceeds 40%. The selection of urban park green space involved the directory of urban parks and an actual investigation. According to the sampling selection, 15 community parks near children's daily living areas were selected, which were widely distributed, as shown in Appendix A.

### Participants

In our study, based on child psychologist Jean Piaget's theory of cognitive development (54) and our previous research protocols (55), children were divided into three age groups: 4–7 years, 8–11 years, and 12–15 years. The field investigations were carried out on the site of children's play activities in urban green spaces. The field research was conducted every weekend in July, which was during the summer vacation period for children. According to a preliminary study, the number of children's activities on weekends was more than that on average working days. Therefore, we randomly set the time for recruiting interviewees to be every Saturday and Sunday in the urban parks. Questionnaires were distributed using sample surveys. Among the children aged 4–7, due to their limited cognitive ability (38), the children and guardians completed the questionnaire together. At the same time, and by trained observers, behavioral characteristics of the children's activities were recorded. After the subjects filled out the questionnaires, they returned them immediately. A total of 300 questionnaires were distributed. After excluding the missing questions and other problems, there were 254 valid questionnaires, and the effective rate of the questionnaires was 83%.

### Measurement procedures

In terms of measurement indicators, the OpenStreetMap platform was used to obtain the park POI data, and the ArcGIS software was used to calculate green space characteristic indicators such as 10-min walkability and the NDVI. Regarding children's play areas, the Woolley and Lowe assessment tool was used to evaluate the spatial and environmental characteristics of children's play space in urban parks (38, 56), as shown in Appendix B.

To assess social anxiety in children, we used the Social Anxiety Scale for Children-Revised (SASC-R) (14) because of its good validity and wide application (15, 57). Three dimensions were included, i.e., fear of negative evaluation (SAD-FNE), social avoidance and distress to novelty (SAD-NEW), and general social avoidance and distress (SAD-GEN). Each adopted item was assessed on a seven-point rating scale from “never” (7) to “always have” (1).

### Green space metrics

To select relevant urban park indicators, we referred to a previous research scheme (27), and to express the characteristics of urban green spaces we included area scale and the normalized difference vegetation index (NDVI) of the urban green spaces. The NDVI further indicated the plant coverage in the urban green spaces. The accessibility of park green space is one of the basic indicators for evaluating service efficiency. Generally, the walking proximity index of an urban green space is used, that is, the appropriate walking distance and walking time. The walking distance or time threshold was based on the law regarding the appropriate distance for an urban green space^1^ or the survey and research results of walking preference time designated in (58–60). In this paper, because the research object was children in urban parks and green spaces, their walking distance to the park was closer to a 10-min walking distance. The accessible area based on a 10-min walk is a specific 10-min walking distance, that is, a distance of about 500 meters that creates a circular buffer zone representing the service area of each corresponding point (61). Therefore, our study used the ArcGIS network analysis and network service area analysis methods to calculate the accessibility coverage area, where the accessibility area of pedestrians in the park referred to the 10-min coverage area.

### Data analysis

We also tested the reliability and validity of the Children's Social Anxiety Scale and the Woolley and Lowe's play space assessment tool. The SPSS software was used to standardize the factors involved in the questionnaire. A reliability analysis was used to study the reliability and accuracy of the quantitative data. First, the alpha coefficient was determined. If this value was higher than 0.8, the reliability was high. The Cronbach's alpha coefficients of the SASC-R and Woolley and Lowe's assessment tool in this study were 0.848 and 0.819, respectively. The KMO and Bartlett tests were used to verify validity. If the KMO value was higher than 0.8, the research data were considered to be suitable for extracting information (excellent validity from the side reaction). The KMO for the SASC-R and Woolley and Lowe's assessment tool were 0.842 and 0.816, respectively. Therefore, the validity of the study data was good, and it could be inferred that the validity and reliability of the questionnaire were reasonable, indicating that the data were accurate and useful. The survey data were analyzed using the SPSS software based on the collection of game activity site assessments and questionnaires for each green space. The Spearman and Pearson correlation tests calculated the relationship between urban green space characteristics and children's social anxiety, the relationship between play activity space characteristics and children's social anxiety, and the relationship between activity characteristics and children's social anxiety. The tests were performed at *p* < 0.01 and *p* < 0.05 to test for significant differences. A stepwise regression analysis was also carried out to test the relationships among particular children's play space elements, environmental elements, and social anxiety in the green space. In addition, an analysis of variance (ANOVA) was used to test for significant differences in children's activity types and social anxiety.

## Results

### Descriptive statistics

The personal attributes and activity characteristics of the children are shown in Table 1. In terms of age, the proportion of the three age groups is similar, among which, the proportion of the 4–7-year-old age group is slightly higher (35.57%) and the proportion of the 12–15-year-old age group is slightly lower (29.90%). This shows that the populations in city parks are relatively equal in age and can accommodate the activity needs of children of different ages. In terms of gender, there is a slightly higher proportion of boys, i.e., 14.44% higher than girls, which may be related to different gender play habits, with boys preferring outdoor activities. In terms of activity frequency, the lowest proportions are less than once a week and more than five times a week; the highest proportion is 3–4 visits a week to a city park. In terms of the length of activities in urban parks, the lowest proportions are shorter than 30 min and more than 120 min, while a common activity duration is between 30 and 120 min, the activity duration between 60 and 90 min accounts for the highest proportion of 39.37%. Regardin activity distance, it can be seen that 1–2 m is the most common distance, accounting for 46.46%. The lowest proportion of activity distances are <0.5 m or more than 4 m, which are 2.36 and 7.48%, respectively. In our study, the most appropriate children's social distance when playing outdoor games was 1–2 m.

**Table 1:** Description of the study samples.

**Item**	**Option**	**Amount**	**Proportion (%)**
Age (years)	4–7	87	34.25
	8–11	91	35.83
	12–15	76	29.92
Gender	Boys	149	58.66
	Girls	105	41.34
Activity frequency (times/week)	<1	8	3.15
	1–2	50	19.69
	3–4	103	40.55
	4–5	78	30.71
	>5	15	5.91
Activity duration (min)	<30	24	9.45
	30–60	56	22.05
	60–90	100	39.37
	90–120	60	23.62
	>120	14	5.51
Activity distance (m)	<0.5	6	2.36
	0.5–1	41	16.14
	1–2	118	46.46
	2–4	70	27.56
	>4	19	7.48

### Results of the relationship between children's social anxiety and green space characteristics

Research Question 1 mainly explores the relationship between urban green space characteristics and social anxiety. In this study, we calculated the relationship between the urban green space index and children's social anxiety, as shown in Table 2. A correlation analysis was used to study the correlations among FNE, SAD-NEW, SAD-G, green space area, 10-min accessibility, and the NDVI. The Spearman correlation coefficient was used to indicate the strength of the correlation. The children's social anxiety factors significantly negatively correlated with the 10-min accessibility and the NDVI. Among them, FNE had the greatest influence on accessibility based on a 10-min walk and the NDVI, and the influence coefficients were −0.227 and −0.354, respectively (*p* < 0.01).

**Table 2:** Correlation results of social anxiety and spatial environment.

	**FNE**	**SAD-NEW**	**SAD-G**
Green space area	−0.117	−0.089	−0.054
10-min accessibility	−0.227^**^	−0.146^*^	−0.159^*^
NDVI	−0.354^**^	−0.281^**^	−0.297^**^

* *p* < 0.05,

** *p* < 0.01.

### The results of the relationships among children's social anxiety and play field characteristics

#### The relationship between spatial environment and social anxiety

To explore the relationships between urban green play spaces and children's social anxiety, a correlation analysis was conducted to study the correlations among FNE, SAD-NEW, SAD-G, spatial, and environmental characteristics. As shown in Table 3, spatial and environmental characteristics both have significant negative relationships with the three factors of social anxiety. Among them, spatial environment significantly influenced FNE, and the correlation coefficient value was −0.489 (*p* < 0.01). Similarly, environmental characteristics also had the greatest impact on FNE, and the correlation value was −0.390 (*p* < 0.01). Moreover, spatial and environmental characteristics both had the least influences on SAD-G.

**Table 3:** Correlation results of social anxiety and spatial environment.

	**FNE**	**SAD-NEW**	**SAD-G**
Spatial characteristics	−0.489^**^	−0.468^**^	−0.452^**^
Environmental characteristics	−0.390^**^	−0.357^**^	−0.346^**^

^*^p < 0.05,

** *p* < 0.01.

#### Spatial elements affecting social anxiety

To further answer Research Question 2, a stepwise regression analysis was performed with 11 spatial features as independent variables and social anxiety as the dependent variable, as shown in Table 4. Finally, the number of remaining immovable facilities, the seats, and the material of the ground are identified as three items in the model, and the R-square value is 0.246, which means that these three elements of spatial characteristics can explain 24.6% of the changes in social anxiety. The stepwise regression model did not include the other eight spatial characteristic variables. In the green space, fixed play equipment has the highest impact on social anxiety (B = −0.250, *p* < 0.01), and surfacing materials have the lowest impact on social anxiety (B = −0.187, *P* < 0.01). Moreover, the model passed the F-test (F = 27.128, *p* = 0.000 < 0.05). Through the regression model analysis, the model formula is: social anxiety = 4.444–0.186 ^*^ fixed play equipment-0.181 ^*^ seats-0.145 ^*^ surfacing materials.

**Table 4:** Stepwise regression results of social anxiety and spatial elements.

	**Unstandardized coefficients**		**Standardized coefficients**	***t***	***p***	**VIF**	**R^2^**	**Adjusted R^2^**	**F**
	**B**	**Standard error**	**Beta**						
Constant	4.444	0.188	-	23.609	0.000^**^	-			
Number of non removable facilities	−0.186	0.045	−0.250	−4.161	0.000^**^	1.195	0.246	0.237	F _(3,250)_ = 27.128, *p =* 0.000
Seats	−0.181	0.048	−0.226	−3.759	0.000^**^	1.196			
Surfacing materials	−0.145	0.047	−0.187	−3.063	0.002^**^	1.229			

Dependent variable: social anxiety.

D-W value: 1.131.

^*^p < 0.05,

** *p* < 0.01.

#### Environmental elements affecting social anxiety

A stepwise regression analysis was performed with five game environmental characteristic elements as independent variables and social anxiety as a dependent variable, as shown in Table 5. Finally, two items, i.e., attractiveness and challenges, are identified in the model, and the R-square value is 0.143, which means that these two environments can explain 14.3% of the changes in social anxiety. In addition, the three environmental features, including visual stimulation, providing learning opportunities, and being suitable for all ages, were not included in the stepwise regression model. The attractiveness and challenge of the environment have similar effects on social anxiety, and the model passed the *F*-test (F = 25.746, *p* = 0.000 < 0.05), indicating that the model is valid. The model formula is social anxiety = 4.099–0.195 ^*^ attractiveness-0.212 ^*^ challenging.

**Table 5:** Stepwise regression results of social anxiety and environmental factors.

	**Unstandardized coefficients**		**Standardized coefficients**	***t***	***p***	**VIF**	**R^2^**	**Adjusted R^2^**	***F***
	**B**	**Standard error**	**Beta**						
Constant	4.099	0.186	-	22.065	0.000^**^	-			
Attractiveness	−0.195	0.050	−0.245	−3.882	0.000^**^	1.208	0.170	0.164	F _(2,251)_ = 25.746, *p* = 0.000
Challenging	−0.212	0.055	−0.245	−3.880	0.000^**^	1.208			

Dependent variable: social anxiety.

D-W value: 1.053.

^*^p < 0.05,

** *p* < 0.01.

### Results of the relationships among social anxiety and activity characteristics in children

#### The relationships among activity characteristics and social anxiety

To answer Research Question 3, a correlation analysis was performed to study the correlations among FNE, SAD-NEW, and SAD-G and the frequency, duration, and distance of outdoor activities, as shown in Table 6. All three assessment factors of social anxiety are negatively related to children's activities. FNE has a significant relationship with activity frequency, duration, and distance; the correlation coefficient values are −0.151, −0.199, and −0.136, respectively. The SAD-NEW items, i.e., activity frequency and distance, both show significant relationships, and the correlation coefficient values are −0.132 and −0.159, respectively. SAD-G only has a significant relationship with activity frequency.

**Table 6:** Correlation results of social anxiety and children's activity.

	**FNE**	**SAD-NEW**	**SAD-G**
Activity frequency	−0.151^*^	−0.132^*^	−0.162^**^
Activity duration	−0.199^**^	−0.126^*^	−0.089
Activity distance	−0.136^*^	−0.159^*^	−0.057

* *p* < 0.05,

** *p* < 0.01.

#### Differences in activity types and social anxiety

A one-way analysis of variance was performed to study the differences in activity types for FNE, SAD-NEW, and SAD-G. Table 7 shows that samples of the different activity types all show significant relationships (*p* < 0.05) with FNE, SAD-NEW, and SAD-G. Social activities, such as playing seesaw and slides, correlate with low social anxiety scores. Rule-based games such as hide-and-seek have relatively high scores for social anxiety. This is followed by functional and imaginative activities. In contrast, social activities score lower on social anxiety. Furthermore, others conduct multiple comparison analyses, as shown in Appendix C. There is a significant difference between social anxiety groups regarding activity type. This is a further answer to Research Question 3.

**Table 7:** Correlation results of social anxiety and children's activity.

	**Activity type (mean** ± **standard deviation)**					***F***	***p***
	**Rule class (*n* = 58)**	**Function class** **(*n* = 87)**	**Social class (*n* = 64)**	**Construction class (*n* = 11)**	**Imagination class (*n* = 43)**		
FNE	3.60 ± 0.68	2.81 ± 0.71	2.33 ± 0.60	2.39 ± 0.66	2.74 ± 0.79	27.895	0.000^**^
SAD-NEW	3.21 ± 0.58	2.79 ± 0.61	2.36 ± 0.63	2.33 ± 0.73	2.56 ± 0.69	16.123	0.000^**^
SAD-G	3.37 ± 0.66	2.82 ± 0.72	2.51 ± 0.65	2.48 ± 0.69	2.74 ± 0.78	12.867	0.000^**^

^*^p < 0.05,

** *p* < 0.01.

## Discussion

First, in this study, we investigated the relationships between children's social anxiety in 15 urban community parks and play space environments and explored the relationships between children's activities and social anxiety under the influence of space. Children's social anxiety was significantly negatively correlated with the NDVI, 10-min accessibility, play space characteristics, and environmental characteristics of urban green spaces. The accessibility of an urban green space is also considered to be one of the essential indicators to measure the efficiency of the design for children (52), and the higher the green space NDVI, the lower the social anxiety of children, which is further proof of the positive benefits of the NVDI (27). The findings also provide empirical evidence for the impact of urban green space planning on the park design–children's social anxiety relationship. Our results can help urban planners and policy makers to better understand what types of urban green spaces and outdoor play spaces can better alleviate children's social anxiety and can affect children's health.

Second, urban community parks are outdoor play spaces that are frequently visited by children (62). In our research, we found that the number of immovable facilities and seats in the play space and the variety of surfacing materials were negatively correlated with children's social anxiety. Especially, these facilities in the play space are more closely related to social interaction (63). These findings provide support for children's outdoor social interactions, activities, and other needs (64). The attractiveness and challenge of environmental features also reduce the level of social anxiety in children (65). Previous studies have shown the importance of natural elements in cities for children's health and activity (66). On this basis, our research shows that, in urban green spaces, the design of the environment and the impact of services on children are also particularly important. The combination of natural and artificial elements may provide greater benefits for children's wellbeing. Extending the time of children's activities, promoting the frequency of children's visits to green space, and promoting children's social activities are effective ways to reduce the level of social anxiety. This is particularly important for early-rise interventions for social anxiety in children (67).

Thirdly, in terms of the influence of children's social anxiety, we found that children engage in many activities in outdoor play space, such as running, jumping, seesaw, and ball games, and these aerobic exercises have been confirmed to reduce social anxiety, which is consistent with the results of our study. At the same time, we also found that the frequency and duration of activity and the distance between children's activities were closely related to social anxiety. Play spaces are more closely related to children's activities (39). It should be pointed out that, in our study, the most common value of the social distance of children in the outdoor play space of urban parks was 1–2 m, which was different from the common social distance of adults, i.e., 1.2–3.5 meters. To explain this difference, we found that some studies have shown that children's social distance is closer than that of adults (68). This also supports the validity of our experimental results. Activities to support this distance are mainly focused on ball games and activities with facilities such as seesaws and slides. In our study, social play was found to be negatively correlated with social anxiety scores. This was consistent with existing manifestations of social anxiety in children; often, the more socially anxious and less accepting of peers that children are, the less likely they are to engage in social play (69). It can be concluded that strengthening children's outdoor activities, and encouraging collectivism and group wellbeing (70) provide a new solution for alleviating children's social anxiety (71), and has positive guiding significance for the design and optimization of children's play spaces in urban parks.

Existing research on children's social anxiety has mostly focused on psychological medicine and social sciences, with a detailed classification of their living environments; major studies have explored the effects of family factors, school factors, and social factors on children's anxiety. The impact of family on children is the most direct and widely researched, and research on the psychological impact of green activity spaces on children is less well documented than adult health research, but should still be an important focus of current research because children are in a period of rapid growth and development and are more sensitive to the effects of natural exposure (72). Most of the studies are about children's psychological depression and anxiety, and there are almost no studies that have specifically focused on the effects of urban park play spaces on children's social anxiety. Our study was conducted from this aspect. It expands on the benefits of urban green spaces in terms of mental health. Our study belongs to the initial exploration of this field.

Finally, the limitations of the current study should be acknowledged. On the one hand, this study is a cross-sectional design, which limits the understanding of the causal relationship between children's play fields in urban green spaces and children's social anxiety and play space-based activities. The current study focused on the relationship between an urban green space and children's health benefits in summer, a relationship that may change with the season. As a result, longitudinal and experimental studies are recommended as future research to establish causal inferences about the effects of year-round children's play places in urban green spaces on children's social anxiety and social interactions. In addition, perhaps due to the limited number of people studied, we cannot derive demographic attributes such as gender differences with children's play space assessment. Additionally, local and social anxiety about children's activities were self-reported. It may have varied due to green space attachment economics and regional differences (73), which may have contributed to the study's findings. Moreover, due to the limitation of sample size, our study cannot be extended to the differences in age and gender of children.

## Conclusions

This study explores the benefits of children's play space in urban green spaces and the relationship between the space environment and children's levels of social anxiety. Based on our findings, several conclusions can be drawn as follows:

First, the NDVI and accessibility of urban green spaces affect children's social anxiety. However, the relationship between the NDVI and children's social anxiety was a more significant degree of correlation. Secondly, among the spatial elements of children's play spaces in green spaces and the model constructed by social anxiety, three elements, i.e., the number of immovable facilities, seats, and surfacing materials, were the most significant. Environmental features significantly associated with children's social anxiety were the attractiveness and challenge of the environment. Finally, children's activity frequency, duration, and distance from each other were all related to social anxiety. This study expects to create a higher-quality outdoor activity environment in urban green spaces for children to play and socialize in public places and to provide valuable help in improving child-friendly cities.

## Data availability statement

The original contributions presented in the study are included in the article/Supplementary material, further inquiries can be directed to the corresponding author/s.

## Ethics statement

The studies involving human participants were reviewed and approved by Jilin Agricultural University Ethics Committee. Written informed consent to participate in this study was provided by the participants' legal guardian/next of kin.

## Author contributions

Conceptualization and writing–review and editing: YB, MG, and DL. Methodology and visualization: MG. Formal analysis: XZ. Investigation and data curation: YB and XZ. Resources and supervision: YB, XZ, and DL. Writing—original draft preparation: YB. Funding acquisition: DL. All authors have read and agreed to the published version of the manuscript.

## Funding

This research was funded by the State Key Program of National Natural Science of China (Grant No. 52238003), the China Postdoctoral Science Foundation (Grant No. 2017M622964), the Chongqing Social Science Foundation (Grant No. 2021NDQN64), and the Research Topics of China Youth Research Association (Grant No. 2022B27).

## Conflict of interest

The authors declare that the research was conducted in the absence of any commercial or financial relationships that could be construed as a potential conflict of interest.

## Publisher's note

All claims expressed in this article are solely those of the authors and do not necessarily represent those of their affiliated organizations, or those of the publisher, the editors and the reviewers. Any product that may be evaluated in this article, or claim that may be made by its manufacturer, is not guaranteed or endorsed by the publisher.

## Footnotes

^1^ https://www.mohurd.gov.cn/gongkai/fdzdgknr/tzgg/201811/20181130_238590.html
