# Supplementary material for: The influence of outdoor play spaces in urban parks on children's social anxiety
Source: Front Public Health. 2022 Dec 6;10:1046399. doi: 10.3389/fpubh.2022.1046399 (PMC9763895; doi:10.3389/fpubh.2022.1046399)
Supplement: Supplementary file 1 [file Data_Sheet_1.docx]

**Appendix A.** Location of sample urban green space.

| 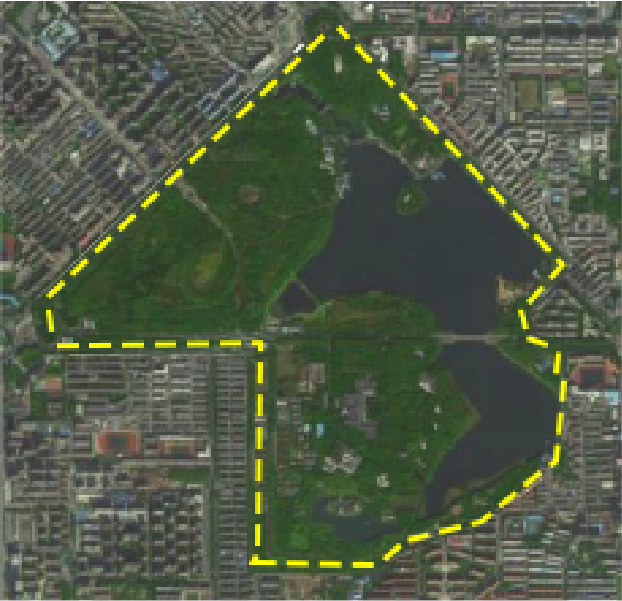  Nanhu Park | 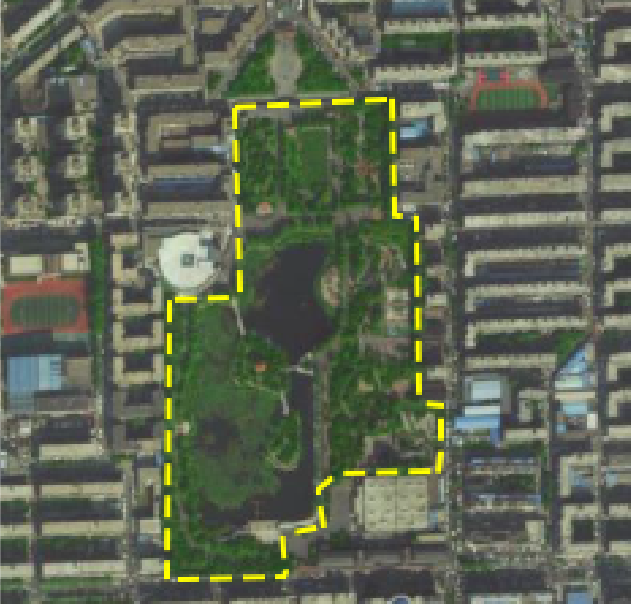  Laodong Park | 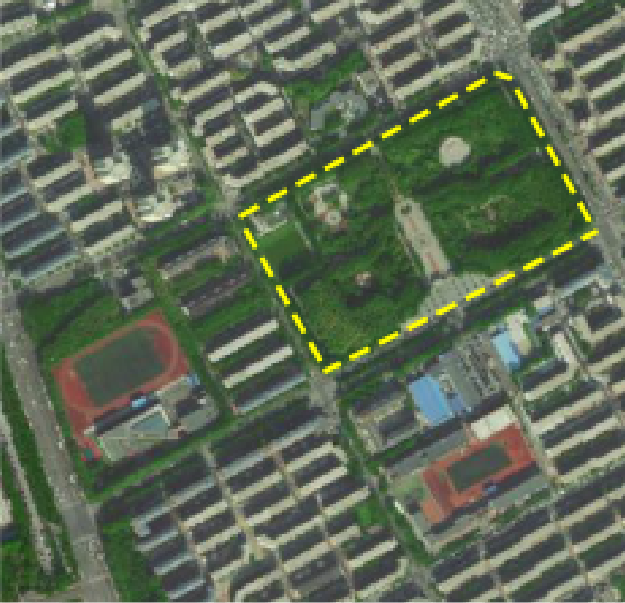  Daishan Park | 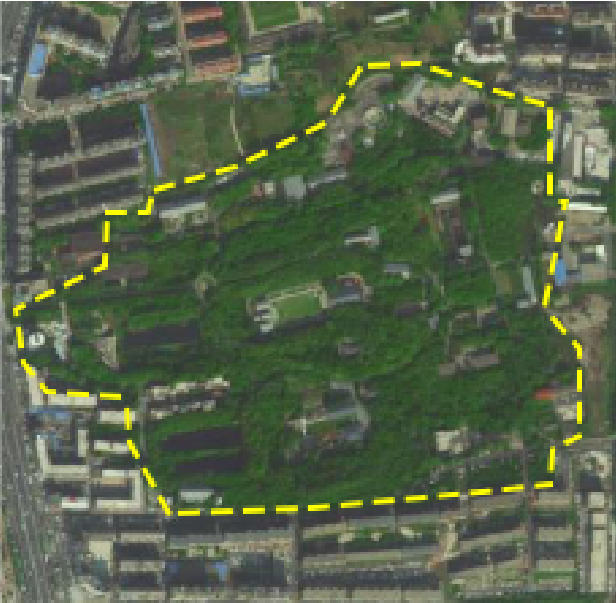Shuiwenhuashengtai Park |
| --- | --- | --- | --- |
| 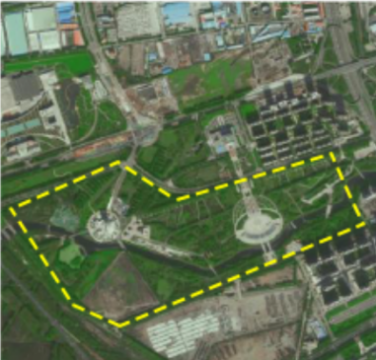  Guojiqiche Park | 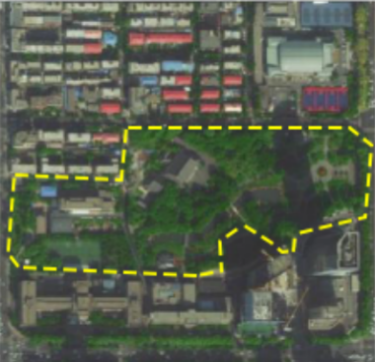  Mudan Park | 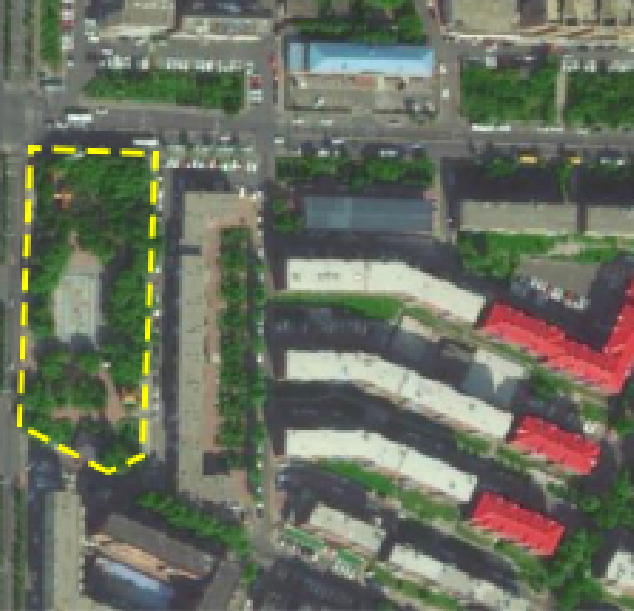  Haishi Park | 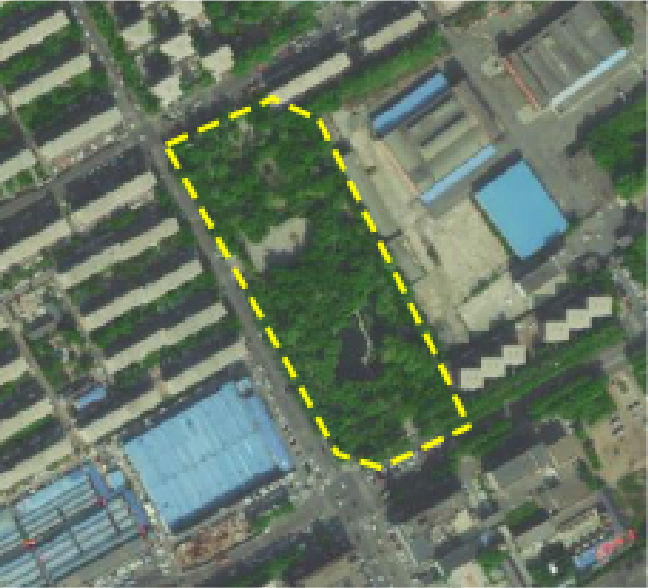  Jinxiu Park |
| 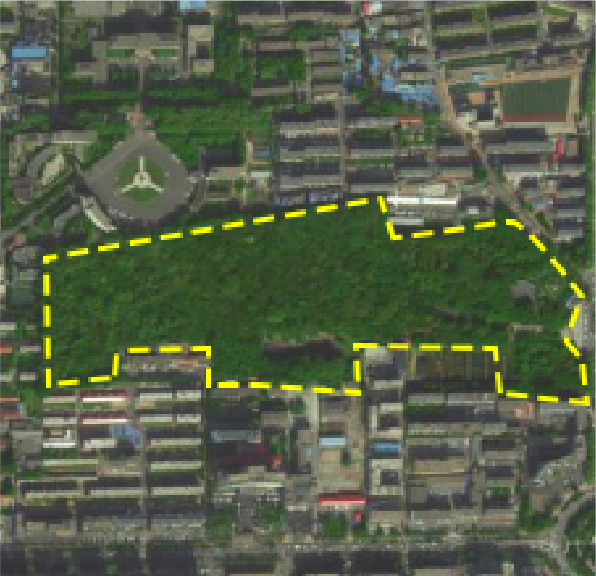  Linyuan Park | 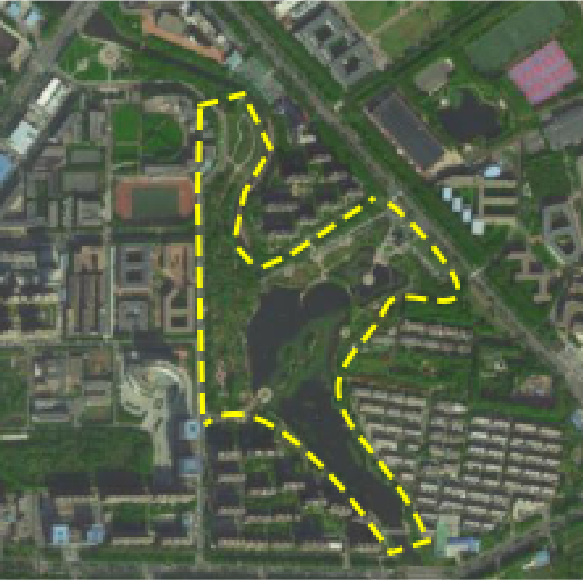  Sanjiahu Park | 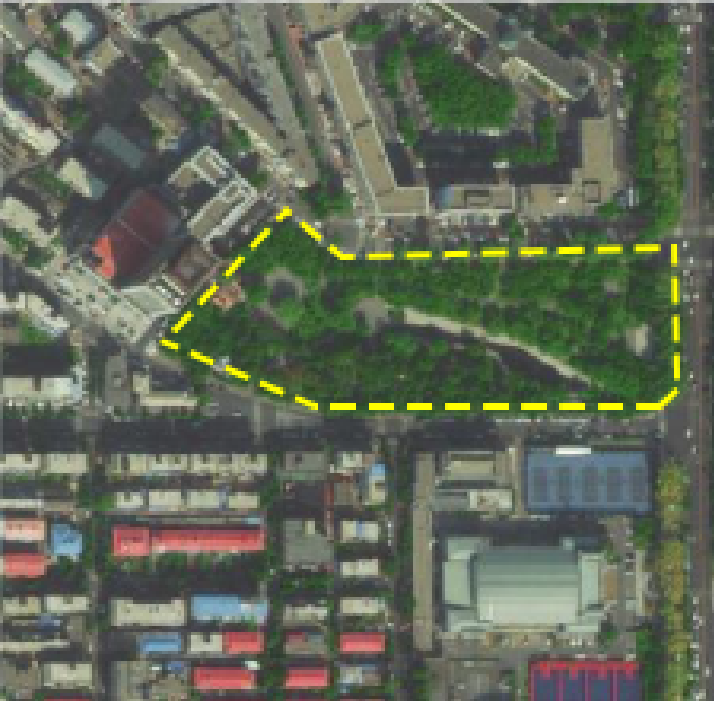  Xinghuacun Park | 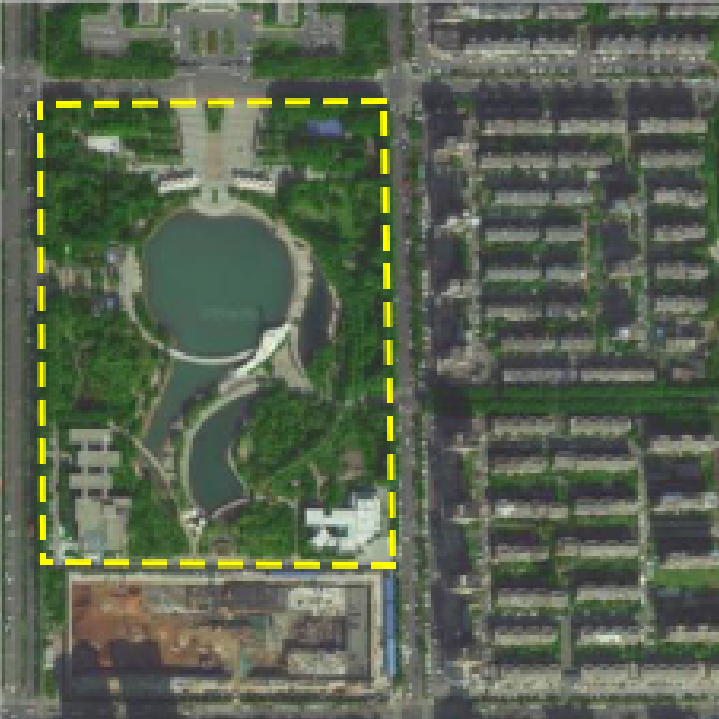  Zhengfuzhongxin Park |
| 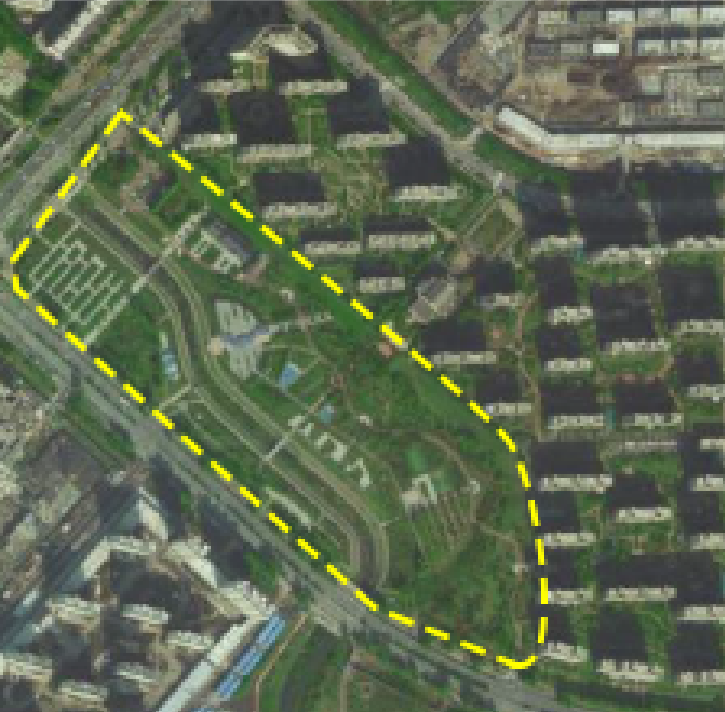  Fuyuhe Park | 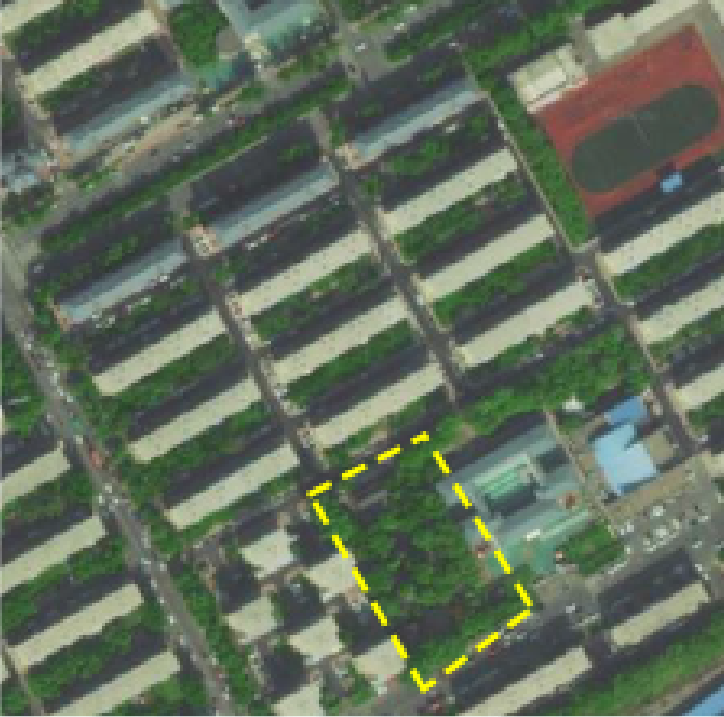  Jincheng Park | 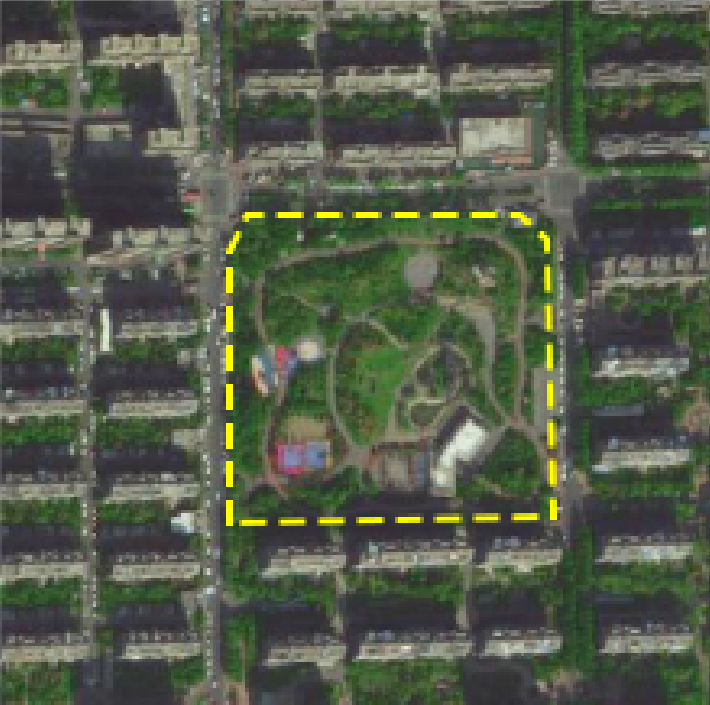  Laoqian Park |  |

**Appendix B.** Woolley & Lowe Evaluation Tool

|  | **Element** | Assignment method |
| --- | --- | --- |
| **Activity types** | **Constructive:**  manipulation and control of the environment | Yes/No |
|  | **Imagery:**  develops fine and gross motor skills, integrating, muscles, nerves and brain functions |  |
|  | **Fantasy:**  exploration of new role and situations through the experimentation of language, concepts, drams and emotion in a risk free environment |  |
|  | **Social:**  interaction with others develops notion  of social rules and responsibility by  sharing and cooperating |  |
|  | **Games with rules:**  develops an awareness of how to react in social situations which are controlled by rules and boundaries |  |
|  | **Element** | **Scoring 0–5** |
| **Spatial**  **characteristics** | **Fixed play equipment:**  Number of pieces of equipment(climbing apparatus, spring mounted, slides, balancing,beams, swings, see-saws, multi play structures) | 0 = none ; 1 = one ; 2 = two-three;3=  four-five; 4= six-seven; 5= contains all of  above |
|  | **Moveable equipment:**  Dependent upon the number of pieces of moveable equipment | 0 = none；1 = one；2 = two pieces；3 = three  pieces；4 = four pieces；5 = five + pieces |
|  | **Open space:**  Dependent upon open space being available for free movement for individual, group and team activities | 0 = none; 1 = limited, physical barriersLimit free movement for individual, group or team activities; 2 = free movement for one of the above; 3 = free movement for two of the above; 4 = some free movement for all of the above; 5 = no physical barriers, free movement for all of the above |
|  | **Different sizes and types of spaces:**  Dependent upon access to very small/private,small, medium, large, sheltered, exposed spaces | 0 = none ; 1 = one; 2 = two; 3 = three; 4 =  four; 5 = all these types of spaces |
|  | **Vegetation/ trees:**  Dependent upon the variety of vegetation:  visual stimulation and opportunities for  interaction | 0 = none; 1 = minimal; 2 = limited types; 3  = several different types in part of  site,visually stimulating or Encourages  interaction; 4 = several different types across site, visually stimulating or encourages interaction; 5 = several different types across  whole or part of site, visually stimulating and  encourages interaction |
|  | **Landform:**  Changes in landform which is stimulating,engaging and challenging | 0 = no changes; 1 = predominantly flat, minimal  of the above; 2 = some changes, minimal of the  above; 3 = several changes, one of the above; 4  = several changes, two of the above; 5 = several  changes, all of the above |
|  | **Movable materials materials:**  Access to, quantity of, and opportunity to move loose materials across the site | 0 = none; 1 = little access; 2 = very small quantities and very small, defined location;3 = small quantities or small defined location; 4 = useable and movable;5 = useable and movable across the whole of the site |
|  | **Water and sand：**  Access to and opportunity to engage and manipulate water and sand |  |
|  | **Natural materials：**  Access to, number of and availability of natural materials across the site such as stones, water, sand, bark,moss, leaves, mud, logs,fruit, sticks | 0 = no access; 1 = one type, across entire site or in areas of the site; 2 = 2–3 types in confined locations; 3 = 2–3 types across entire site; 4 = 4+ types in confined locations within site; 5 = 4+ types across entire site |
|  | **Obvious physical boundaries：**  Existence of clear and rigid boundary and visual stimulation and engagement such as fencing | 0 = whole/part of site defined by physical boundary, neither visually stimulating or engaging; 1 = whole of site defined by physical boundary, visually stimulating or engaging; 2 = whole of site defined by physical boundary, visually stimulating and engaging; 3 = part of site defined by physical boundary, visually stimulating or engaging; 4 = part of site defined by physical boundary, visually stimulating and engaging; 5 = whole site is free from rigid physical boundaries |
|  | **Seats:**  Quantity and location of seating opportunities | 0 = none; 1 = some, not within play area; 2 = limited within play area, located around the edge; 3 = limited within the play area, isolated & sporadic; 4 = some throughout the site, does not encourage children to interact; 5 = large amount throughout the play area |
|  | **Surfacing materials:**  The type of material and whether it is attractive and stimulating including grass, sand, bark, gravel, rubber。 | 0 = none; 1 = one, not engaging or stimulating; 2 = one or two types, engaging or stimulating; 3 = one or two types, engaging and stimulating; 4 = more than two types, engaging and stimulating; 5 = 3+ types, engaging and stimulating. |
|  | **Element** | **Scoring 0–5** |
| **Environmental Characteristics** | Is the area enticing? | 0 = no evidence; 1 = contains one of the above; 2 = contains two of the above; 3 = contains three of the above; 4 = contains four of the above; 5 = contains all of the above |
|  | Is the area stimulating by creating a range of experiences, containing  natural elements and allowing for movement? | 0 = no evidence; 1 = contains one of the above; 2 = contains two of the above; 3 = contains three of the above; 4 = contains four of the above; 5 = contains all of the above |
|  | Is the area challenging? | 0 = no evidence; 1 = contains one of the above; 2 = contains two of the above; 3 = contains three of the above; 4 = contains four of the above; 5 = contains all of the above |
|  | Are there learning  opportunities? | 0 = no evidence; 1 = limited opportunities to interact with materials or the natural environment and does not allow for any kind of manipulation or experimentation; 2 = access to a few types of materials, but limited access to the natural environment, and allows for either manipulation or experimentation; 3 = access to a few types of materials, some access to the natural environment, and allows for either manipulation or experimentation on one area of the site; 4 = access to a large range of materials, the natural environment and allows for manipulation and experimentation in more than one area of the site; 5 = access to a large range of materials and the natural environment and allows for manipulation and experimentation across the whole site without restrictions |
|  | Is the area available for all age groups？ | 0 = no evidence; 1 = caters for one age group; 2 = caters for two age groups; 3 = caters for three age groups; 4 = caters for four age groups;5 = caters for all age groups |

**Appendix C.**. Multiple comparison results

|  | (I)Name | (J)Name | (I)Average value | (J)Average value | Average value difference(I-J) | p |
| --- | --- | --- | --- | --- | --- | --- |
| FNE | Rule class | Function class | 3.603 | 2.812 | 0.792 | 0.000** |
|  | Rule class | Social class | 3.603 | 2.334 | 1.269 | 0.000** |
|  | Rule class | Construction class | 3.603 | 2.386 | 1.217 | 0.000** |
|  | Rule class | Imagination class | 3.603 | 2.735 | 0.868 | 0.000** |
|  | Function class | Social class | 2.812 | 2.334 | 0.478 | 0.000** |
|  | Function class | Construction class | 2.812 | 2.386 | 0.425 | 0.055 |
|  | Function class | Imagination class | 2.812 | 2.735 | 0.076 | 0.583 |
|  | Social class | Construction class | 2.334 | 2.386 | -0.052 | 0.816 |
|  | Social class | Imagination class | 2.334 | 2.735 | -0.401 | 0.006** |
|  | Construction class | Imagination class | 2.386 | 2.735 | -0.349 | 0.145 |
| SAD-NEW | Rule class | Function class | 3.213 | 2.791 | 0.421 | 0.000** |
|  | Rule class | Social class | 3.213 | 2.359 | 0.853 | 0.000** |
|  | Rule class | Construction class | 3.213 | 2.333 | 0.879 | 0.000** |
|  | Rule class | Imagination class | 3.213 | 2.563 | 0.650 | 0.000** |
|  | Function class | Social class | 2.791 | 2.359 | 0.432 | 0.000** |
|  | Function class | Construction class | 2.791 | 2.333 | 0.458 | 0.023* |
|  | Function class | Imagination class | 2.791 | 2.563 | 0.228 | 0.073 |
|  | Social class | Construction class | 2.359 | 2.333 | 0.026 | 0.899 |
|  | Social class | Imagination class | 2.359 | 2.563 | -0.203 | 0.127 |
|  | Construction class | Imagination class | 2.333 | 2.563 | -0.229 | 0.292 |
| SAD-G | Rule class | Function class | 3.366 | 2.819 | 0.547 | 0.000** |
|  | Rule class | Social class | 3.366 | 2.508 | 0.859 | 0.000** |
|  | Rule class | Construction class | 3.366 | 2.477 | 0.889 | 0.000** |
|  | Rule class | Imagination class | 3.366 | 2.735 | 0.631 | 0.000** |
|  | Function class | Social class | 2.819 | 2.508 | 0.311 | 0.007** |
|  | Function class | Construction class | 2.819 | 2.477 | 0.342 | 0.127 |
|  | Function class | Imagination class | 2.819 | 2.735 | 0.084 | 0.553 |
|  | Social class | Construction class | 2.508 | 2.477 | 0.031 | 0.893 |
|  | Social class | Imagination class | 2.508 | 2.735 | -0.227 | 0.125 |
|  | Construction class | Imagination class | 2.477 | 2.735 | -0.258 | 0.287 |
| * p<0.05 ** p<0.01 | | | | | | |
